# Seasonal differences of corticosterone metabolite concentrations and parasite burden in northern bald ibis (*Geronticus eremita*): The role of affiliative interactions

**DOI:** 10.1371/journal.pone.0191441

**Published:** 2018-01-24

**Authors:** Verena Puehringer-Sturmayr, Claudia A. F. Wascher, Matthias-Claudio Loretto, Rupert Palme, Mareike Stoewe, Kurt Kotrschal, Didone Frigerio

**Affiliations:** 1 Core Facility Konrad Lorenz Forschungsstelle for Behaviour and Cognition, University of Vienna, Grünau im Almtal, Austria; 2 Department of Behavioural Biology, University of Vienna, Vienna, Austria; 3 Department of Biology, Anglia Ruskin University, Cambridge, United Kingdom; 4 Department of Cognitive Biology, University of Vienna, Vienna, Austria; 5 Department of Biomedical Sciences, Unit of Physiology, Pathophysiology and Experimental Endocrinology, University of Veterinary Medicine, Vienna, Austria; University of Pretoria, SOUTH AFRICA

## Abstract

The reproductive season is energetically costly as revealed by elevated glucocorticoid concentrations, constrained immune functions and an increased risk of infections. Social allies and affiliative interactions may buffer physiological stress responses and thereby alleviate associated effects. In the present study, we investigated the seasonal differences of immune reactive corticosterone metabolite concentrations, endoparasite burden (nematode eggs and coccidian oocysts) and affiliative interactions in northern bald ibis (*Geronticus eremita*), a critically endangered bird. In total, 43 individually marked focal animals from a free-ranging colony were investigated. The analyses included a description of initiated and received affiliative interactions, pair bond status as well as seasonal patterns of hormone and endoparasite levels. During the reproductive season, droppings contained parasite eggs more often and corticosterone metabolite levels were higher as compared to the period after reproduction. The excretion rate of endoparasite products was lower in paired individuals than in unpaired ones, but paired animals exhibited higher corticosterone metabolite concentrations than unpaired individuals. Furthermore, paired individuals initiated affiliative behaviour more frequently than unpaired ones. This suggests that the reproductive season influences the excretion patterns of endoparasite products and corticosterone metabolites and that affiliative interactions between pair partners may positively affect endoparasite burden during periods of elevated glucocorticoid levels. Being embedded in a pair bond may have a positive impact on individual immune system and parasite resistance.

## Introduction

The reproductive season is considered socially and energetically costly when compared to the post-reproductive one [1,2]. Such costs of reproduction are often manifested by up-regulated glucocorticoid secretion and constrained immune functions [3,4]. For instance, territorial male Alpine chamois (*Rupicapra rupicapra*) showed elevated cortisol metabolite concentrations and parasite levels during the mating season as compared to the post-mating period [5]. Chronically elevated glucocorticoid levels suppress immune function [6] and the down-regulation of the immune system may increase the susceptibility to parasitic infections [7–10]. The physiological costs of parasitic infection can be confirmed by experimentally removing parasites. Wild mice populations (*Peromyscus maniculatus* and *P*. *leucopus;* [11]) and cliff swallows (*Petrochelidon pyrrhonota;* [12]) showed reduced corticosterone levels when intestinal nematodes or hematophagous ectoparasites were removed. Furthermore, in greylag geese (*Anser anser*) parasite excretion decreased throughout the parental season, which has been suggested to be related to the high energetic costs of the reproductive season [13]. Furthermore, in altricial birds parental care is modulated by the offspring’s parasite load [14,15]. When caring for parasitized offspring, blue tit parents (*Parus caeruleus*) increased their feeding rates in compensation [14], whereas reduced parental effort was shown in male spotless starlings (*Sturnus unicolor*) with parasitized nests [15]. However, information on the contribution of other individual life-history traits (e.g. sex, age, social status) is difficult to obtain in free-living social animals, which may complicate the analyses of parasite infection patterns [13,16].

Social allies and affiliative behaviour may help buffering physiological stress responses [17–21]. For example, Barbary macaques (*Macaca sylvanus*; [22]) and wild chimpanzees (*Pan troglodytes schweinfurthii*, [23]) showed decreased glucocorticoids in the company of a bonding partner, suggesting that affiliative interactions may provide a buffer against stressors, benefitting immune function and alleviating parasite burden. Furthermore, in chicks of domestic hens *(Gallus gallus domesticus*), the presence of the mother buffered the stress response to an aversive stimulus [24] and in greylag geese the presence of socially supportive family members reduced corticosterone secretion [13,18,19]. Although individual levels of corticosterone metabolites in parental geese were negatively correlated with the number of offspring [19], the excretion of nematode eggs increased with family size [13], indicating that parental effort is costly [25]. A recent study in northern bald ibis (*Geronticus eremita*) showed that paired individuals excreting high levels of endoparasite products during the reproductive season also engaged in more allopreening behaviour [26].

We presently aim at investigating the seasonal differences of concentrations of excreted immune reactive corticosterone metabolites (CM), parasite burden (as measured by the number of samples containing endoparasite products) and frequencies of affiliative behaviour between the reproductive and post-reproductive season in the free-roaming and individually marked colony of northern bald ibis at the Konrad Lorenz Research Station (Austria). This seasonally monogamous and year-round colonial species breeds in dense colonies and forages in flocks, that often split into subgroups of different sizes [27]. Pair bond stability changes with time in seasonally monogamous species, with strong social bonds among the adults at the start of each reproductive season and loose ones during the post-reproductive period [28]. Especially for paired individuals we expected higher levels of corticosterone metabolites and endoparasite products in the reproductive period as compared to the post-reproductive one. We also expected paired individuals to engage more often in affiliative interactions than unpaired ones, with interactions occurring more frequently during the reproductive season than outside.

We distinguished between initiated and received affiliative behaviour since they may differ in their effects on glucocorticoid secretion, as measured by a reduced excretion of CM [29], self-directed behaviours (self-grooming/self-scratching) [30,31] or heart rate [32–34]. We predicted that receiving rather than initiating affiliative behaviour may lead to a greater reduction of glucocorticoid levels and hence to reduced parasite burden.

## Material and methods

This study complies with all current Austrian laws and regulations concerning the work with wildlife. Catching, restraining of the birds, observing the animals and collecting droppings were performed under Animal Experiment Licence Number 66.006/0026-WF/V/3b/2014 by the Austrian Federal Ministry for Science and Research (EU Standard, equivalent to the Animal Ethics Board). We confirm that the owner of the land, the Duke of Cumberland, gave permission to conduct the study on this site. All data were collected non-invasively. Birds were habituated to the presence of humans.

### Field site and study animals

The study was conducted in Grünau im Almtal (Upper Austria, 47°48’E, 13°56’N). The free-ranging colony of northern bald ibis was established in 1997 at the Konrad Lorenz Research Station (KLF) in Grünau im Almtal by hand-raising zoo-bred chicks [35] in coordination with the European Breeding Programme (EEP, [36]). Since 2001 the birds raise their chicks autonomously and the colony has grown to more than 40 individuals. The animals are housed in a large aviary, which is open year round, at the local Herzog-von-Cumberland game park. They are free-flying and roam the foraging grounds in the Almtal-region, returning to the aviary for roosting at night and for breeding. From November to May the birds are supplied twice a day with hash made from 1-day-old chicks mixed with soaked dog food (Alpha Multicroc, RWA Raiffeisen Ware Austria AG, Vienna, Austria). All birds are individually marked with a combination of coloured leg rings. At the start of data collection, the colony consisted of 43 birds (focal animals; N_males_ = 24, N_females_ = 19). The age ranged from 0 to 16 years (mean: 4.2). According to the classification described by Böhm and Pegoraro [27] focal individuals were assigned to three different age classes: (1) juvenile (first year after hatching, N = 6), (2) sub-adult (second and third year after hatching, N = 15) and (3) adult (from fourth year on, N = 22). Details about focal individuals (name, sex, year of hatching, pair bond status) are given in S1 Table.

### Data collection

Behavioural data and individual droppings were collected over a period of 112 days between 15 May 2015 and 31 October 2015. Days of data collection were irregularly distributed over the sampling period, as the northern bald ibis population flew to Molln (Upper Austria, 47°53’E, 14°15’N; 25 km linear distance from Grünau im Almtal; 112 days in Grünau and 76 days in Molln) and stayed there from August until October. In Molln, standardised data collection was not possible, due to inaccessible foraging grounds (e.g. cow paddocks). Hence, data collected in Molln were excluded from the analyses. We included both the reproductive and post-reproductive season in this study. The reproductive season was determined as the time from rearing to fledging of the offspring (generally from May to mid-June, provisioning phase), whereas the post-reproductive season lasted from mid-June to October. Due to individual variations in the start of egg-laying, beginning and end of both periods were determined separately for each breeding pair depending on the hatching date of the offspring.

#### Behavioural data

The behaviour was measured by applying a continuous recording method [37,38] for each focal individual. All focal observations were evenly distributed during daylight hours (i.e. between 08.00 AM and 08.00 PM) to avoid temporal biases. Behavioural protocols lasted 5 minutes per individual and were recorded using the software CyberTracker (CyberTracker Conservation, Cape Town, South Africa; www.cybertracker.org, [39]). The frequencies of affiliative behaviours initiated and received by the focal birds, such as greeting, preening, preening invitation, mutual bill shaking and contact sitting were monitored in the aviary or the nearby foraging grounds (for a detailed description of northern bald ibis’ ethogram: see [40]). In sum, a total of 284 behavioural protocols were collected over the entire period ($\bar{x}$ ± SE: 6.6 ± 0.8 focal observations per individual). The mean frequencies per minute per individual per week were: initiated– 0.72 ± 0.10, received– 0.10 ± 0.04.

#### Collection of droppings and analysis of corticosterone metabolites

Droppings of the focal individuals were collected in order to determine the amount of (1) CM and (2) excreted endoparasite products (nematode eggs and coccidian oocysts).

Steroid metabolites in droppings represent an integrated, proportional record of the unbound portion of plasma steroids depending on the frequency of defecation and gut passage time [41]. We assumed the gut passage time of northern bald ibises to be 2–3 hours, comparable to the white ibis (*Eudocimus albus)* which are similar in size and diet [42]. The sample collection was performed daily independently of behavioural observations. To account for possible endogenous diurnal variations, droppings were collected during the same time period (04.00 PM to 07.00 PM CET). The samples were collected immediately after defecation in Eppendorf® microtubes (Eppendorf®, Hamburg, Germany), with one sample per tube in order to avoid contamination with other droppings. Due to constraints of dropping collection in the field, the sample size varies in the different analyses. The samples were (1) stored on ice during collection and then frozen at -20°C within 3 hours after collection for the determination of CM or (2) stored at +6°C and analysed within 7 days after collection for parasite burden. A total number of 142 droppings were collected for CM analysis (on average: $\bar{x}$ ± SE = 3.74 ± 0.47 samples per individual) and 130 for parasite burden (on average: $\bar{x}$ ± SE = 4.06 ± 0.57 samples per individual). The different sample sizes result from the fact that CM and parasite samples were collected independently from each other. CM were determined using an enzyme immunoassay (EIA, selection for the best suited assay see below) [43] at the laboratory of the Department of Behavioural Biology, University of Vienna (Austria). The intra- and interassay coefficients of variation were below 5%.

#### Selection of the best-suited assay

Besides the data collection described above, we compared several available enzyme immunoassays in order to select the best-suited one for measuring corticosterone secretion in the northern bald ibis, a necessary step when using such non-invasive methods for measuring faecal glucocorticoid metabolites [44,45].

**Fig 1:**
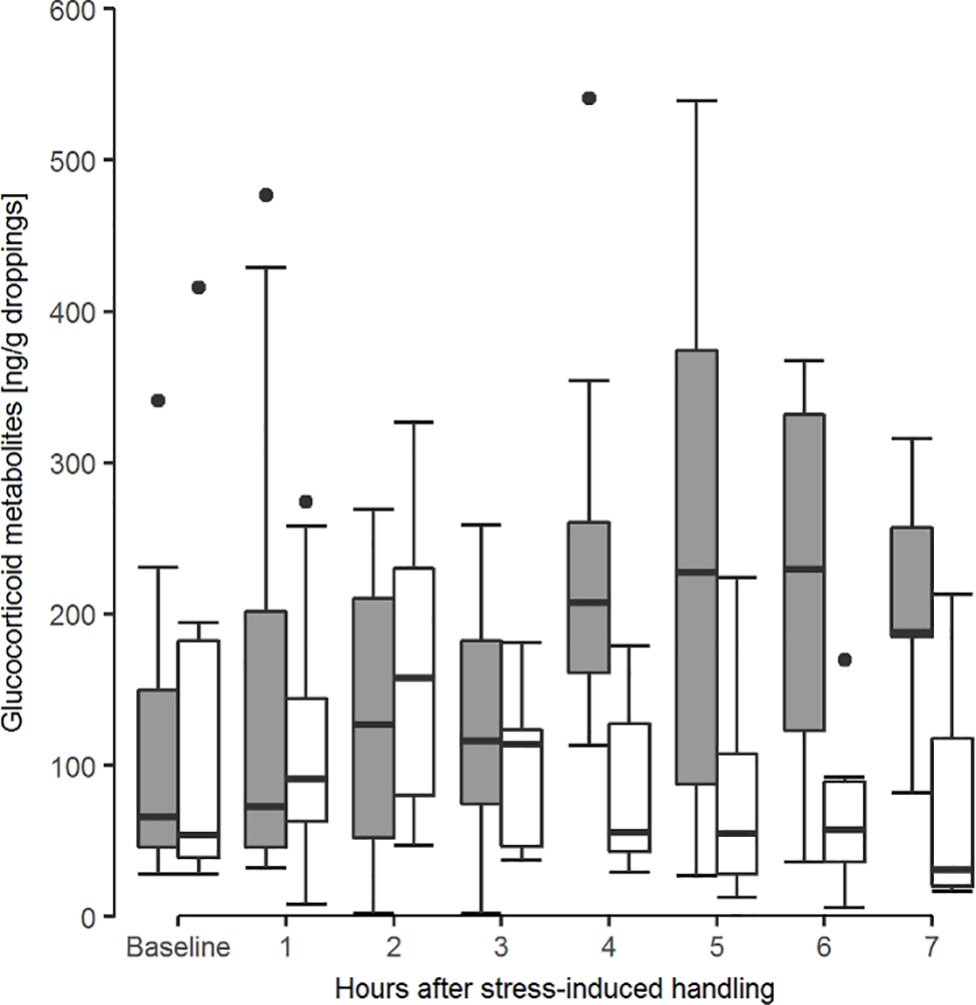
Detected corticosterone metabolite concentrations for two assays. Median (± SE) levels of excreted corticosterone metabolites of all four individuals for the 11-oxoaetiocholanolone enzyme immunoassay (best-suited assay, grey boxplots) and the corticosterone enzyme immunoassay (white boxplots). N_individuals_ = 4, N_samples_ = 81.

To evaluate which of the currently available EIAs is best suited for measuring adrenocortical activity of the northern bald ibis, a handling experiment was run on two experimental days in December 2015 and January 2016. Four individuals (2 males and 2 females, S1 Table) were subjected to a handling stress. For baseline CM levels droppings were collected one day prior to the experiment during the same time period in which an increase in CM concentrations was expected in response to the handling stress on the following day (10.00 AM to 12.00 AM). We tested males and females separately on two different days. The focal individuals were captured between 09.30 AM and 09.45 AM on both experimental days to avoid the early morning peaks of adrenocortical activity [46] and to prevent unnecessary disturbance of the other colony individuals. Upon capture, each bird was put into a cloth bag (approximately 40x45x2 cm) for 10 minutes. Afterwards, the individuals were released into a test room, which was divided into two compartments by a mesh. Both birds were in visual and acoustic contact with each other and with the other members of the colony in the main aviary. We kept the birds to be tested separated from the other birds to prevent cross-contamination of droppings and to enable the simultaneous data collection of two individuals. The test room was enriched with branches and the floor was covered with a clear tarp to facilitate the collection of each dropping. Dropping collection was conducted for 6 hours after the beginning of the experiments (i.e. approximately between 10.00 AM and 04.00 PM). The exact time of defecation was recorded for each sample. An aliquot (0.5 g) of each dropping was extracted with 5 ml 60% methanol [47]. The analysis was run at the Department of Biomedical Sciences, Unit of Physiology, Pathophysiology and Experimental Endocrinology, University of Veterinary Medicine in Vienna (Austria). The following 5 assays, which work well in diverse bird species, were tested on northern bald ibis droppings to determine the best-suited one: a corticosterone EIA ([41]; this assay has previously been used for northern bald ibis droppings by Sorato and Kotrschal [48]), an 11-oxoaetiocholanolone EIA [43], a cortisone EIA [49], an 11ß-hydroxyaetiocholanolone EIA [50] and a 5α-pregnane-3ß,11ß,21-triol-20-one EIA [51]. All extracts were diluted with assay buffer (1:10) and 50 μl were used for the EIA. All samples were analysed in duplicates. The 11-oxoaetiocholanolone assay (‘best assay’) turned out to be the most appropriate for northern bald ibis droppings, meaning that this assay is considerably more sensitive compared to the others. In fact, it detected higher peak values within the same sample (highest increase of median CM concentration from baseline–best assay: 348%, corticosterone EIA: 293%; Fig 1). Furthermore, the ‘best assay’ also detected peaks of CM concentrations over a longer time period after the stress-induced handling, whereas the corticosterone EIA only detected the first peak concentration.

#### Parasitological examination

The examination for excreted parasite products (nematode eggs and coccidian oocysts) was done using a flotation method in a McMaster counting chamber [52]. At least 0.1 g dropping was diluted with the triple volume of saturated NaCl solution (350 g NaCl, 1000 ml distilled water) and filtered through a sieve to remove large food particles and debris. The remaining solution was transferred into both McMaster counting chambers, in which the excreted coccidian oocysts and nematode eggs were counted. A value for oocysts/eggs per gramme faeces was calculated according to Hiepe et al. [52]:

$$eggs/oocysts\;per\;gramme=\frac{number\;of\;counted\;eggs*volume\;of\;salt\;solution\;(ml)}{volume\;of\;counting\;chambers\;(0.30\;ml)*weight\;of\;dropping\;(g)}$$

Due to generally low parasite egg and oocyst abundance in the samples (percentage of samples containing nematode eggs: 22.52%; percentage of samples containing coccidian oocysts: 15.32%), we treated samples either as containing or not containing nematode eggs or coccidian oocysts.

### Statistical analyses

All statistical analyses were conducted using R version 3.2.5 [53] and the additional packages ‘glmmADMB’ [54,55] for calculation of generalised linear mixed models (GLMM) and ‘MuMIn’ [56] for information-theoretic model selection as well as model averaging based on the information criteria. A ratio per minute was calculated for all behavioural parameters observed. Due to few occurrences of behavioural interactions, we treated initiated and received affiliative behaviour as a binomial variable, i.e. occurred or not occurred events, which were taken into further statistical analyses.

To assess which factors influence (1) parasite burden (samples containing nematode eggs and coccidian oocysts–yes/no), (2) initiated (yes/no) and (3) received (yes/no) affiliative behaviour (all three response variables) we used GLMMs with binomial error distribution and logit link function. To analyse which factors influence (4) CM (response variable) we used a GLMM with gamma error distribution and inverse link function. Fixed factors in these models were (1) sex, (2) age, (3) pair bond status (paired, unpaired) and (4) season (reproductive and post-reproductive season). Since the northern bald ibis is a social, seasonally monogamous and colonial species, affiliative interactions can be observed between all colony members. Therefore, we also included pair bond status as a fixed factor in the affiliative behaviour models. Subject identities were included as random factors in all GLMMs to control for between-subject variation and unbalanced design. Field constraints did not allow a regular data collection for the three data sets (parasite burden, CM, affiliative behaviour). Interactions between fixed factors were not considered in the statistical analyses as they did not improve the model fit.

To select the best models, we used an information theoretic approach and calculated all possible candidate models [57]. We ranked them according to their AICc values (second-order form of Akaike’s information criterion to account for small sample sizes; [58]) and selected the models with ΔAICc ≤ 2 with respect to the top-ranked model. The models were averaged in order to create model-averaged coefficients following Burnham & Anderson [57].

## Results

**Fig 2:**
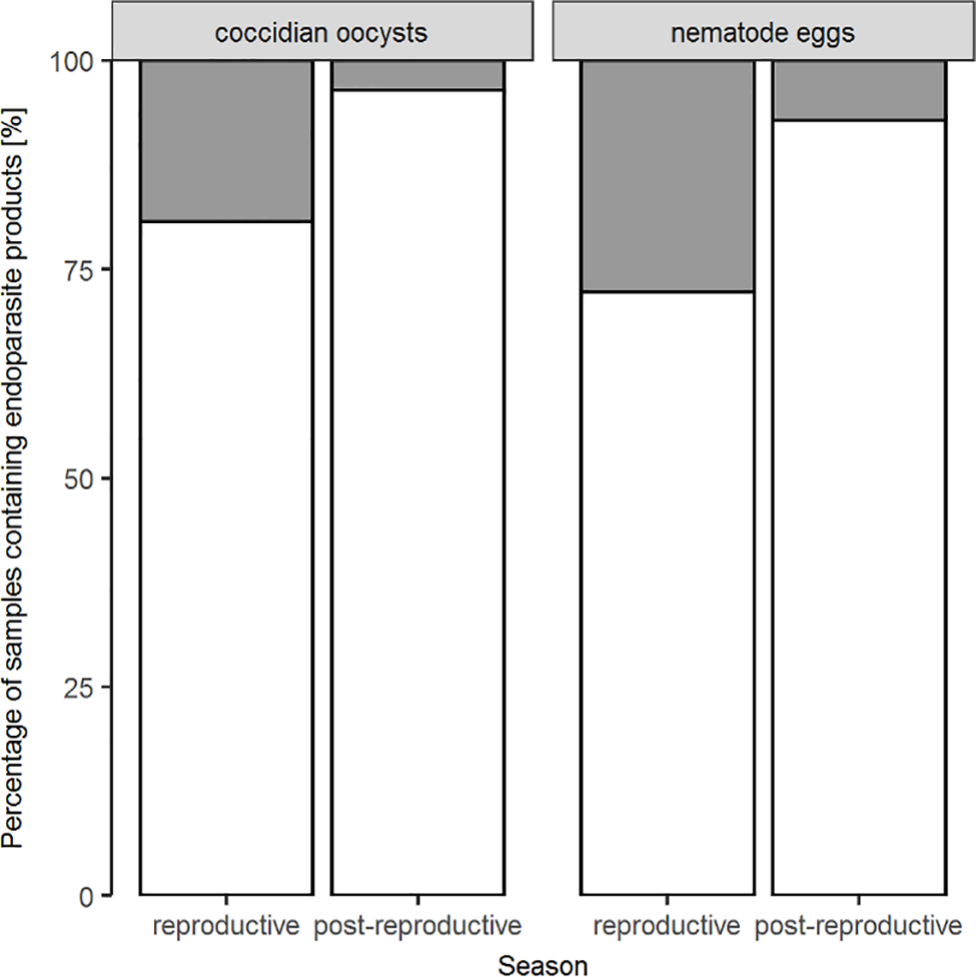
Seasonal differences in excretion patterns of endoparasite products. Percentage of samples containing (grey bars) as well as not containing (white bars) endoparasite products for the reproductive and post-reproductive season. N_reproductive_ = 83, N_post-reproductive_ = 28, N_samples_ = 111.

**Fig 3:**
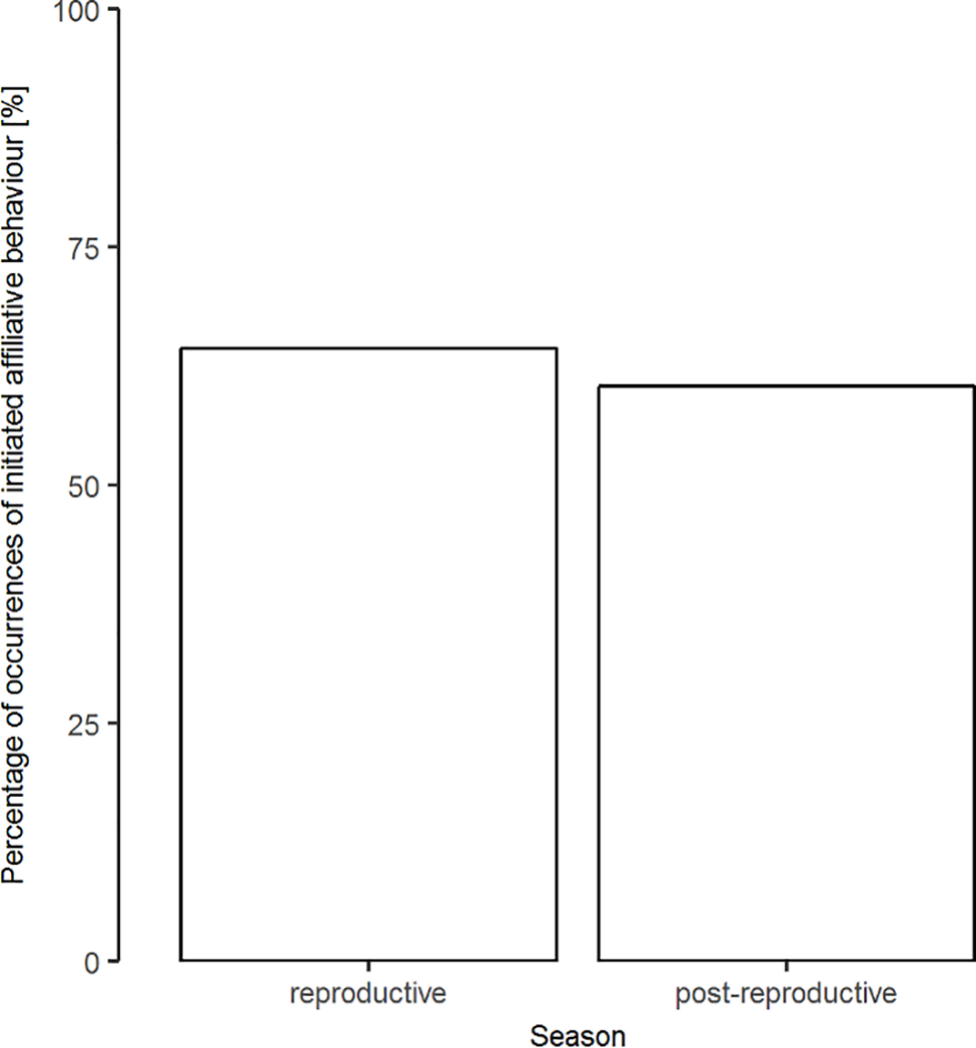
Seasonal differences in engaging in affiliative interactions. Percentage of occurrences of initiated affiliative behaviour for the reproductive and post-reproductive season. N_reproductive_ = 87, N_post-reproductive_ = 197.

Model-averaged results identified season as the strongest determinant of the excretion patterns of nematode eggs and coccidian oocysts. The number of droppings containing endoparasite products was highest during the reproductive season as compared to the time after reproduction (Fig 2). While season was the second-most important variable for corticosterone metabolite (CM) concentrations, it was the least important parameter for initiated affiliative behaviour. During the reproductive season, we found higher CM levels as compared to the post-reproductive period. Affiliative interactions were initiated more frequently during the reproductive season, while the rates decreased in the post-reproductive period (Fig 3).

**Fig 4:**
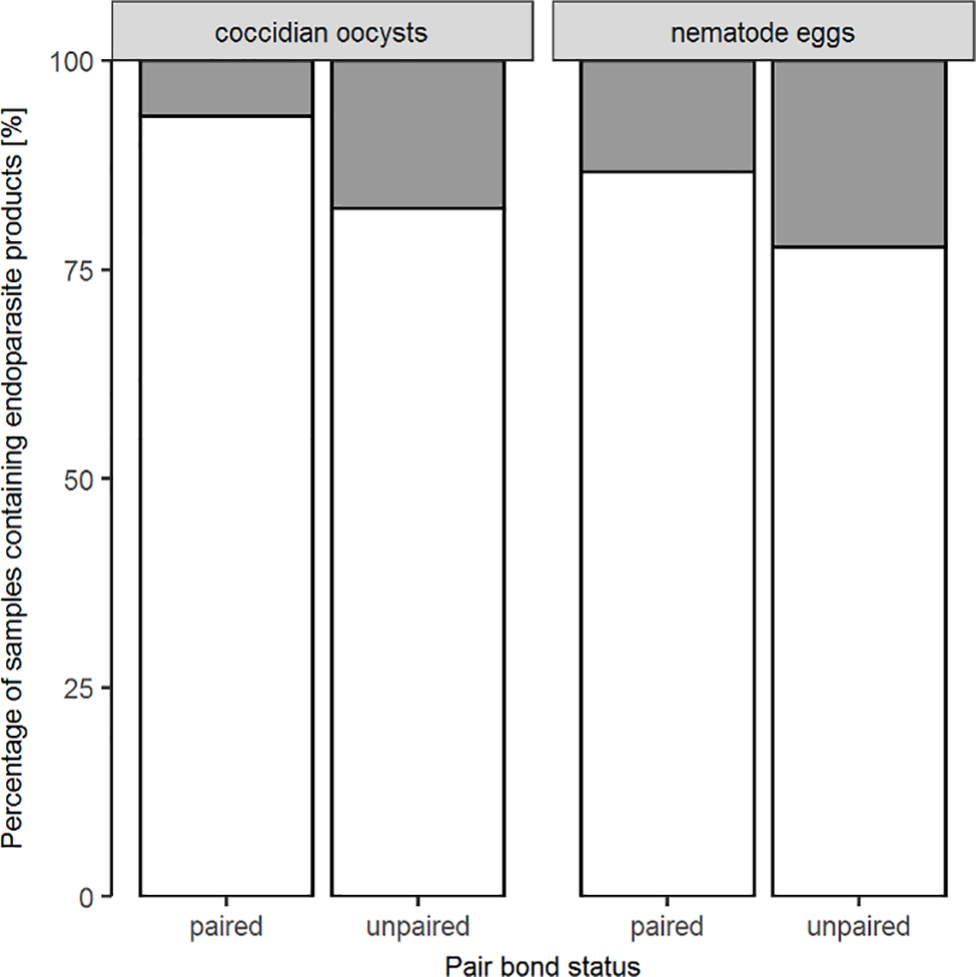
Differences in excretion patterns of endoparasite products depending on pair bond status. Percentage of samples containing (grey bars) as well as not containing (white bars) endoparasite products for paired and unpaired individuals. N_paired_ = 10, N_unpaired_ = 22, N_samples_ = 130.

Pair bond status was the second-most important variable influencing the excretion of coccidian oocysts. Furthermore, pair bond status was indicated as the third-most important variable for nematode egg excretion, CM levels and initiated affiliative behaviour. Paired individuals excreted fewer droppings containing endoparasites (Fig 4) and exhibited generally higher CM concentrations and initiated more often affiliative behaviour as compared to unpaired ones.

We found that initiated affiliative behaviour and the excretion pattern of nematode eggs were best explained by sex. Males initiated affiliative interactions more frequently and excreted a higher number of droppings containing endoparasite products than females. The relative importance indicated the variable sex to be less important for coccidian oocysts. Furthermore, sex had no influence on CM.

The most influential variable on the excretion patterns of CM was age, with juveniles exhibiting higher CM levels compared to adults, whereas sub-adult individuals showed lower concentrations than adults. Furthermore, it was the second-most influential variable for initiated affiliative behaviour. A higher frequency of initiating affiliative behaviour was found in juveniles compared to adults, whereas sub-adults initiated less than adults. Age had the least importance when considering the excretion patterns of coccidian oocysts, with lower excretion rates in juvenile and sub-adult individuals as compared to adult ones. Age, however, did not predict the excretion rate of nematode eggs.

All candidate models with received affiliative behaviour as response variable did not improve penalised model fit over the null model, as assessed by AICc, indicating that variation in the data cannot be explained by any of these factors [57].

Statistical details of the top-ranked models are presented in Tables 1 and 2.

**Table 1:** Top-ranked models. Given are the predictors influencing the response variables nematode eggs, coccidian oocysts and initiated affiliative behaviour.

Explanatory variables of each model	Df	logLik	ΔAICc	weight
**nematode eggs**				
season, sex	4	-55.779	0.00	0.712
season, sex, pair bond status	5	-55.599	1.81	0.288
**coccidian oocysts**				
season, pair bond status, sex	5	-47.226	0.00	0.188
season, pair bond status	4	-48.454	0.29	0.163
season, sex	4	-48.501	0.38	0.155
pair bond status, sex	4	-48.599	0.58	0.141
sex	3	-49.973	1.20	0.103
season, pair bond status, age	6	-46.850	1.45	0.091
season	3	-50.204	1.66	0.082
pair bond status, age	5	-48.110	1.77	0.078
**corticosterone metabolites**				
age	5	-704.961	0.00	0.513
age, season	6	-704.466	1.19	0.282
age, pair bond status	6	-704.784	1.83	0.205
**initiated affiliative behaviour**				
sex	3	-285.876	0.00	0.179
sex, age	5	-183.842	0.06	0.174
sex, pair bond status	4	-184.959	0.22	0.160
age	4	-185.083	0.47	0.142
sex, age, season	6	-183.666	1.80	0.073
sex, season	4	-185.794	1.89	0.070
sex, age, pair bond status	6	-183.742	1.95	0.068
age, season	5	-184.787	1.95	0.068
sex, pair bond status, season	5	-184.803	1.98	0.067

Df–degrees of freedom; logLik–log-likelihood; ΔAICc–differences of the second order Akaike information criterion between the best model and the other top-ranked models; weight–Akaike weight.

**Table 2:** Model-averaged coefficients. Given are the coefficients with adjusted standard errors, lower and upper confidence intervals and relative importance of the top-ranked models.

Parameter (*levels*)	Estimate	Adjusted SE	CI lower limit (2.5%)	CI upper limit (97.5%)	Relative importance
**nematode eggs**					
Intercept	-4.71	1.29	-7.23	-2.18	
season (*reproductive period*)	1.65	0.80	0.07	3.22	1.00
sex (*male*)	2.01	1.12	-0.18	4.20	1.00
pair bond status (*unpaired*)	0.11	0.39	-0.86	1.65	0.29
**coccidian oocysts**					
Intercept	-3.84	1.22	-6.23	-1.44	
season (*reproductive period*)	0.85	0.88	0.32	2.83	0.68
pair bond status (*unpaired*)	0.75	0.78	-0.22	2.50	0.66
sex (*male*)	0.94	1.14	-0.52	3.72	0.59
age (*juvenile*)	-2.66	2067.99	-9976.96	9945.44	0.17
age (*sub-adult*)	-0.26	0.73	-3.69	0.66	0.17
**corticosterone metabolites**					
Intercept	4.24	0.19	3.87	4.60	
age (*juvenile*)	0.25	0.43	-0.60	1.10	1.00
age (*sub-adult*)	-0.66	0.29	-1.24	-0.09	1.00
season (*reproductive period*)	-0.04	0.10	-0.42	0.14	0.28
pair bond status (*unpaired*)	-0.04	0.16	-0.80	0.43	0.21
**initiated affiliative behaviour**					
Intercept	0.31	0.27	-0.22	0.84	
sex (*male*)	0.46	0.35	0.01	1.15	0.79
age (*juvenile*)	0.25	0.55	-0.87	1.83	0.52
age (*sub-adult*)	-0.35	0.42	-1.36	0.03	0.52
pair bond status (*unpaired*)	-0.09	0.21	-0.88	0.27	0.29
season (*reproductive period*)	0.04	0.16	-0.38	0.70	0.28

SE–standard error; CI–confidence interval.

## Discussion

We found different modulation patterns of excreted immune reactive corticosterone metabolites (CM) and endoparasites in the reproductive season as compared to the post-reproductive period in the seasonally monogamous but year-round social northern bald ibis. The reproductive season is energetically costly in terms of egg-laying, incubation and rearing of the offspring [59], which was indicated by high CM concentrations during the reproductive period and decreasing CM afterwards. This was mirrored by the decrease of the excretion of endoparasite products as well. In contrast, initiating affiliative behaviour was only weakly modulated by season.

As our data are correlative, we can only speculate about a potential causality between elevated CM concentrations and endoparasite burden. We suggest that the increased excretion of endoparasite products as well as high corticosterone metabolite concentrations during the reproductive season may be the result of a trade-off between reproductive effort and immune function [3]. High CM levels may suppress immune responses [60], impairing the defence against parasites. This would explain the similar excretion patterns of endoparasite products and CM during the reproductive and post-reproductive season. Alternatively, we cannot exclude that patterns of endoparasite burden may have also been driven by the phenology of the parasites themselves, meaning that the seasonality of the parasites and their underlying dissemination could result in certain patterns in parasite product excretion, independent of the animals’ immune response [13,61,62]. However, as parasitic infections are often associated with a suppressed immune system [7,8,10] and CM showed similar secretion patterns, parasite phenology alone is probably insufficient to explain the observed patterns.

We further found that males produced more endoparasite-positive droppings than females, suggesting that particularly the male immune system was affected by social investment. This is also indicated by the fact that males in general, independently of age, initiated more affiliative interactions compared to females, which would be expected to function as social support buffering females glucocorticoid secretion. As males are the donors of social support, which benefits the females, they also may generally be more susceptible to infections with parasites due to reduced immune functions as compared to females [60,63]. Another possible explanation for these sex-specific differences may be the immunosuppressive effects of testosterone [64], whereas oestrogens are thought to enhance immune function [65]. However, Sorato and Kotrschal [48] showed that excreted testosterone metabolite levels are similar between male and female northern bald ibis, indicating that testosterone alone may not be the major driving factor in parasite infection. Hence, patterns of parasite burden may be influenced by several factors, such as pair bond status, reproductive state and rearing condition [66] as well as individual factors such as sex and age [65] and they may be seen as a result of the interaction of all the factors mentioned above [13].

Higher CM levels and excretions of endoparasite products were found in adults compared to sub-adult individuals and adults also initiated more affiliative interactions. This may be linked to sexual maturity and hence to the necessary behavioural and physiological investment into reproduction. As glucocorticoids are the major hormones regulating metabolism [67], this investment is reflected by a greater glucocorticoid up-regulation during the reproductive season as compared to the post-reproductive period [59,68,69]. In contrast, juvenile individuals exhibited higher CM concentrations while having a lower parasite burden than adults and initiated more frequently affiliative interactions. A possible explanation for these age differences may be that age causes changes in the hypothalamic-pituitary-adrenal axis [70]. For instance, plasma corticosterone concentrations in response to a stressor decreased with age in Florida scrub-jays (*Aphelocoma coerulescens*), while the oldest individuals exhibited again greater corticosterone levels [71].

Probably due to relaxed pair bond relationships outside the reproductive season in the northern bald ibis [27], affiliative interactions generally occurred more frequently during the reproductive as compared to the post-reproductive season. Affiliative interactions during the post-reproductive season may be seen as social investment towards old or new pair partners before the start of the new breeding season [27]. As paired individuals exhibited lower excretion patterns of coccidian oocysts compared to unpaired ones, which may indicate that pair partners, via mutual social support, can control endoparasite burden in stressful periods, such as the reproductive season.

Contrary to expectations, we found no link between receiving affiliative behaviour and the excretion of CM and endoparasite products. However, the excretion patterns of endoparasite products were still lower in paired than unpaired individuals. This may indicate that initiating affiliative interactions with social partners may up-regulate immune functions, even though high CM levels are still present. In fact, pair bonded northern bald ibis excreted higher concentrations of CM compared to unpaired ones. Moreover, long-term monogamous avian species, such as greylag geese, show decreased excretion of CM when family members are present during stressful situations [19,72]. In separated zebra finch pairs (*Taeniopygia guttata*) for example, elevated corticosterone concentrations returned to baseline levels after reunion with the pair partner [73]. This implies that familiar individuals are more effective for social buffering than unfamiliar ones [23,74,75]. Similarly in mammals, male Wistar rats (*Rattus norvegicus*) did not show fear-related behaviours, such as freezing, when smelling the odour of a familiar conspecific [75] and squirrel monkey (*Saimiri sciureus*) females showed decreased basal cortisol levels in the presence of a female pair partner [76].

On the other hand, it might be expected that interacting with parasitized conspecifics increase the risk of infection [77]. However, paired individuals were less parasitized with coccidian oocysts than unpaired ones. Furthermore, as being paired is also related to age and dominance [78,79], dominant individuals could defend better resting places, which are less contaminated with faeces.

A previous study on northern bald ibis showed that more nematode eggs were excreted in females than in males during the reproductive season [26], whereas in the present study it was the other way round. Contrary to this previous study, we did not include the egg-laying phase in the data collection, which may be the reason for not finding an elevated excretion of endoparasite products in females. This suggests that the high energetic costs of egg-laying suppress female immune responses in a socially stressful situation. A possible explanation may be that resources are allocated to reproduction during the egg-laying period and hence down-regulate female immune functions [80], which may lead to an increase in parasite burden.

We conclude that excretion patterns of endoparasite products and CM concentrations differ in the colonial and seasonally monogamous northern bald ibis according to season. Even though reproduction is energetically costly and may be accompanied by elevated glucocorticoid concentrations [1,2], affiliative interactions may buffer endoparasite burden during stressful periods. This suggests that being well embedded in a pair bond may have a positive impact on individual parasite burden and therefore also on the immune system, at least for females.

## Supporting information

S1 Dataset Initiated and received affiliative behaviour for single individuals in the reproductive and post-reproductive season. (CSV)

S2 Dataset Corticosterone metabolite (CM) concentrations for single individuals in the reproductive and post-reproductive season. (CSV)

S3 Dataset Excretion patterns of endoparasite products (coccidian oocysts and nematode eggs) for single individuals in the reproductive and post-reproductive season. (CSV)

S1 Table List of all focal individuals. Name, sex (m = male, f = female), year of hatching, age class, pair bond status as well as involvement in the assay comparison are indicated.
(DOCX)
