# Supplementary material for: Seasonal differences of corticosterone metabolite concentrations and parasite burden in northern bald ibis (Geronticus eremita): The role of affiliative interactions
Source: PLoS One. 2018 Jan 24;13(1):e0191441. doi: 10.1371/journal.pone.0191441 (PMC5783627; doi:10.1371/journal.pone.0191441)
Supplement: S1 Table — Name, sex (m = male, f = female), year of hatching, age class, pair bond status as well as involvement in the assay comparison are indicated. (DOCX) [file pone.0191441.s004.docx]

**Table S1. List of all focal individuals.** Name, sex (m=male, f=female), year of hatching, age class, pair bond status as well as involvement in the assay comparison are indicated.

| **Name** | **Sex** | **Year of hatching** | **Age class** | **Breeder** | **Assay comparison** |
| --- | --- | --- | --- | --- | --- |
| Othello | m | 1999 | adult | yes | X |
| Hera | m | 1999 | adult | no |  |
| Hombre | m | 2002 | adult | yes | X |
| Abraxas | m | 2002 | adult | no |  |
| Aleppo | f | 2006 | adult | yes | X |
| Simon | m | 2006 | adult | yes |  |
| Loki | f | 2006 | adult | yes |  |
| Shannara | m | 2007 | adult | yes |  |
| Cian | m | 2008 | adult | no |  |
| Schreckse | f | 2008 | adult | yes | X |
| North Face | m | 2009 | adult | no |  |
| Sequoia | f | 2009 | adult | yes |  |
| Hilda | m | 2009 | adult | no |  |
| Heidi | m | 2010 | adult | no |  |
| Rob | m | 2010 | adult | no |  |
| Balu | m | 2010 | adult | yes |  |
| Paco | m | 2010 | adult | no |  |
| Ozzy | m | 2010 | adult | no |  |
| Tiffi | m | 2011 | adult | no |  |
| Lukas | m | 2012 | adult | no |  |
| Hermine | f | 2012 | adult | yes |  |
| Kurt | m | 2012 | adult | no |  |
| Kleopatra | f | 2013 | sub-adult | no |  |
| Khan | m | 2013 | sub-adult | no |  |
| Elvis | m | 2013 | sub-adult | no |  |
| Minerva | f | 2013 | sub-adult | no |  |
| Oniichan | m | 2014 | sub-adult | no |  |
| Luzifer | m | 2014 | sub-adult | no |  |
| Luna | f | 2014 | sub-adult | no |  |
| Chicco | f | 2014 | sub-adult | no |  |
| Diablo | f | 2014 | sub-adult | no |  |
| Kira | f | 2014 | sub-adult | no |  |
| Mocha | f | 2014 | sub-adult | no |  |
| Quinto | f | 2014 | sub-adult | no |  |
| Sino | f | 2014 | sub-adult | no |  |
| Taska | f | 2014 | sub-adult | no |  |
| Woody | f | 2014 | sub-adult | no |  |
| Aurelia | f | 2015 | juvenile | no |  |
| Azzurro | m | 2015 | juvenile | no |  |
| Sandro | f | 2015 | juvenile | no |  |
| Saphira | f | 2015 | juvenile | no |  |
| Simba | m | 2015 | juvenile | no |  |
| Smirne | m | 2015 | juvenile | no |  |
